# Danshensu attenuated lipopolysaccharide‐induced LX‐2 and T6 cells activation through regulation of ferroptosis

**DOI:** 10.1002/fsn3.3065

**Published:** 2022-09-15

**Authors:** Changting Wang, Zhiming Su, Jian‐Hua Xu, Chih‐Yuan Ko

**Affiliations:** ^1^ Department of General Surgery The Second Affiliated Hospital of Fujian Medical University Quanzhou China; ^2^ Department of Tumor Surgery The Second Affiliated Hospital of Fujian Medical University Quanzhou China; ^3^ Department of Clinical Nutrition The Second Affiliated Hospital of Fujian Medical University Quanzhou China; ^4^ School of Public Health Fujian Medical University Fuzhou Fujian China

**Keywords:** Danshensu, ferroptosis, hepatic stellate cells, liver fibrosis, reactive oxygen species

## Abstract

Liver fibrosis and cirrhosis are primarily caused by the activation of hepatic stellate cells (HSCs), regardless of their etiology. Collagen type I (collagen I) and connective tissue growth factor (CTGF) is produced more readily by activated HSCs. Consequently, identifying the molecular and cellular mechanisms responsible for HSCs activation is essential to better understand its mechanism of action and therapeutic potential. Cell death is caused by iron‐dependent lipid peroxidation during ferroptosis. Ferroptosis plays an important role in the survival of activated HSCs and could contribute to the development of innovative prevention and treatment strategies for liver fibrosis. Danshensu (Dan) is a pure molecule extracted from the *Salvia miltiorrhiza* herb that protects against liver damage. However, Dan's effect on attenuating HSCs activation by regulating ferroptosis remains unclear. The results of this study indicated that lipopolysaccharide (LPS)‐induced LX‐2 and T6 cells activation occurs through the upregulation of collagen I, CTGF, Gpx4, and SLC7A11. Interestingly, Dan attenuated LPS‐induced liver fibrosis in those cells by upregulating collagen I, CTGF, Gpx4, and SLC7A11 and by increasing lipid reactive oxygen species accumulation. Furthermore, the results also showed that the ferroptosis inhibitor liproxstatin attenuated the overproduction of lipid reactive oxygen species in LPS‐activated LX‐2 cells. We conclude that Dan attenuates LPS‐induced HSC activation during liver fibrosis by regulating ferroptosis in LX‐2 and T6 cells.

## Graphical abstract

Danshensu (Dan) attenuates lipopolysaccharide‐induced hepatic stellate cells activation via decreasing expression of collagen І, connective tissue growth factor, glutathione peroxidase 4, cystine/glutamate antiporter xCT, and lipocalin‐2. However, Dan increases lipid reactive oxygen species accumulation.

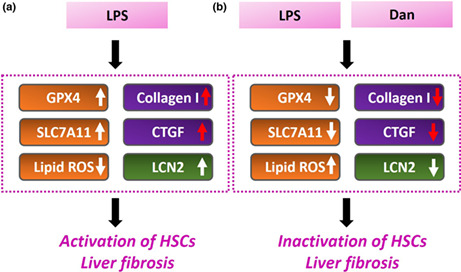

## 1 INTRODUCTION

It is worthwhile to study liver fibrosis extensively because it is a condition that results in progressive liver damage and even failure (Chan et al., 2020). Excessive production of extracellular matrix proteins, including collagen, causes liver fibrosis. Liver fibrosis causes cirrhosis, hepatitis, and portal hypertension and often requires surgery at the end of the disease (Bataller & Brenner, 2005). Fibrogenesis in the liver is characterized by the transition from quiescent hepatic stellate cells (HSCs) to activated, proliferating, migrating, and fibrogenic cells (i.e., myofibroblasts) (Hou & Syn, 2018). Various cellular and molecular signals are involved in the activation of HSCs (Tsuchida & Friedman, 2017). Therefore, by eliminating the HSCs which activate liver fibrosis, it can be resolved.

Treatment with traditional Chinese medicine might be beneficial for liver fibrosis (Li, 2020; Luo et al., 2022). Danshensu (Dan) is a pure molecule derived from the root of the *Salvia miltiorrhiza* herb, *Danshen*. It has a clearly defined chemical structure and demonstrates antioxidative, antiapoptotic, vasodilating, inflammation‐regulating, and lipid‐lowering effects (J. Zhang et al., 2019). Dan appears to exert protective effects by reducing lipid peroxidation, collagen accumulation, and JAK2‐STAT3 signaling, at least partially, in both the CCl4‐induced hepatic fibrosis rat model and TGF‐β1‐induced HSC‐T6 cells (Cao et al., 2019). Dan protects liver tissue from CCl4‐induced cytotoxicity by reducing lipid peroxidation and collagen accumulation, enhancing antioxidant capabilities, as well as inhibiting intrahepatic JAK/STAT signaling pathways involved in collagen homeostasis (Qu et al., 2014). It remains unclear, however, whether Dan attenuates HSCs activation through ferroptosis and endoplasmic reticulum stress.

Ferroptosis is a regulated cell death characterized by the accumulation of toxic lipid hydroperoxides by iron‐dependent mechanisms (Stockwell et al., 2020). The pathological process of ferroptosis results in cell death in degenerative diseases, cancer, stroke, intracerebral hemorrhage, ischemia–reperfusion injury, and kidney disease in mammals (Stockwell et al., 2017). Lipocalin‐2 (LCN2) is a secreted glycoprotein that is associated with neutrophil gelatinase. In neutrophil granules, it was initially identified as a key regulator of cell proliferation, innate immunity, metabolism, apoptosis, tumor metastasis, and liver fibrosis (Chen et al., 2020; Jaberi et al., 2021). In abundantly expressed acute and chronic liver injury models, LCN2 plays a protective role (Asimakopoulou et al., 2016). Based on the results of a previous study, LCN2 massive hepatic expression may increase fibrosis and portal hypertension, both major predictors of mortality and morbidity in alcoholics (Chen et al., 2020). Taken together, the downregulation of LCN2 in activated HSCs may alleviate the progression of liver fibrosis.

In this study, we examined whether Dan attenuates lipopolysaccharide (LPS)‐induced activation of HSCs in liver fibrosis by regulating ferroptosis in LX2 and T6 cells.

## 2 MATERIALS AND METHODS

### 2.1 Cell culture

LX‐2 human and T6 rat HSCs were purchased from Sigma‐Aldrich. Cells were grown in Dulbecco's modified Eagle medium (Gibc) containing 2% fetal bovine serum (Gibco) and 1% penicillin/streptomycin (Gibco) and incubated at 37°C with 5% CO_2_. Furthermore, we used passages 3–8 of cells in this study.

### 2.2 Protein extraction and western blot analysis

Cell protein extraction was performed using radioimmunoprecipitation assay lysis buffer containing a protease inhibitor cocktail (Roche) and phosphatase inhibitor cocktail (Roche), with a reaction time of 30–60 min on ice. A BCA assay kit (Thermo Fisher Scientific) was used to measure the protein concentration in each protein sample.

Western blot analysis was performed using an SDS‐polyacrylamide gel electrophoresis system and then transferred to PVDF membranes of 0.45‐μm pore size. The membranes were blocked with 5% milk or bovine serum albumin (Sigma), placed in 1× TBST buffer for 30–60 min, and incubated with primary antibodies overnight at 4°C. Anti‐β‐actin (#4967, 1:1000 dilution, Cell Signaling), anti‐SLC7A11 (#12691, 1:1000 dilution, Cell Signaling), anti‐Gpx4 (#59735, 1:1000 dilution, Cell Signaling), anti‐collagen І (#72026, 1:1000 dilution, Cell Signaling), anti‐CTGF (#A11067, 1:1500 dilution, ABclonal), and anti‐LCN2 (sc‐365970, 1:500 dilution, Santa Cruz Biotechnology) antibodies were used for probing. On alternate days, the membranes were washed using 1× TBST buffer for 10 min, which was repeated three times. Sections were then incubated with horseradish peroxidase (HRP)‐conjugated secondary antibodies for 60 min at room temperature. The membranes were washed using 1× TBST buffer for 10 min, which was repeated three times. All cell protein levels were analyzed using Cytiva Amersham ECL Prime western blotting detection reagent (Thermo Fisher Scientific) and a ChemiDocTM XRS+S system (Bio‐Rad Laboratories). The intensities of the protein bands were analyzed using ImageJ software.

### 2.3 Lipid reactive oxygen species (ROS) analysis

BODIPY 581/591 C11 (lipid peroxidation sensor) reagents (Thermo Fisher Scientific, Inc.) were used to measure the generation of lipid ROS generation according to the manufacturer's instructions. Briefly, cells were seeded in a cell culture plate 24 h after drug treatment. Cells were incubated with BODIPY 581/591 C11 (lipid peroxidation sensor) reagent for a minimum of 30 min at 37°C in a 5% CO_2_ atmosphere incubator and harvested by centrifugation. After resuspension in 1000 μl 1× PBS buffer, the analysis was performed using a Coulter Cytomic FC 500 flow cytometer (Beckman) and Cell Quest software.

### 2.4 Statistical analysis

Data are expressed as mean ± SEM. Statistical analyses of all data were performed using GraphPad Prism software (version 5.0; USA), and a *p* < .05 was considered statistically significant. Multiple groups were analyzed using a one‐way analysis of variance followed by Tukey's post hoc test.

## 3 RESULTS

### 3.1 LPS‐activated HSCs activation and downregulated Gpx4 and SLC7A11 in LX‐2 cells

**FIGURE 1:**
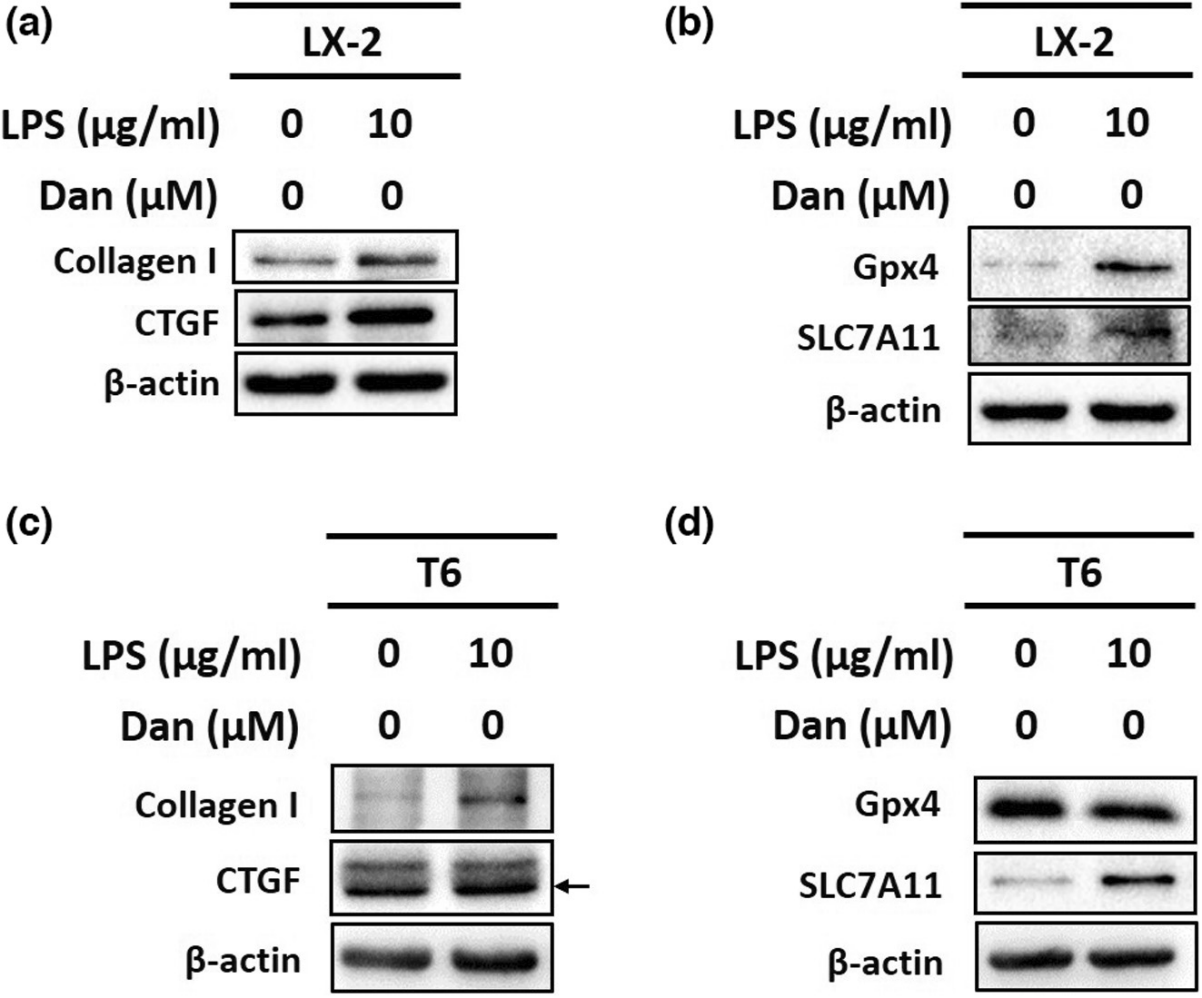
Effect of lipopolysaccharide (LPS) on LX‐2 and T6 cells activation and expression of Gpx4 and SLC7A11. (a) Changes in the protein levels of collagen I and connective tissue growth factor (CTGF) after treatment with LPS in LX‐2 cells. (b) Changes in the protein levels of Gpx4 and SLC7A11 after treatment with LPS in LX‐2 cells. β‐actin is indicated as an internal control. (c) Changes in the protein levels of collagen I and CTGF after treatment with LPS in T6 cells. (d) Changes in the protein levels of Gpx4 and SLC7A11 after treatment with LPS in T6 cells. β‐actin is indicated as an internal control

HSC activation was characterized by the production of collagen I and CTGF in the presence of LPS (Higashi et al., 2017; Martín‐Vílchez et al., 2008; Tsuchida & Friedman, 2017). Our results indicated that LPS (10 μg/ml) increased the expression of collagen I and CTGF in LX‐2 cells (Figure 1a) and T6 cells (Figure 1c). Meanwhile, we detected the levels of the indicated proteins by western blotting to study the activation of HSCs and whether they affect the expressions of Gpx4 and SLC7A11. The results showed that the upregulated expression of Gpx4 and SLC7A11 was detected with 10 μg/ml LPS‐treated LX‐2 cells (Figure 1b) and T6 cells (Figure 1d). These findings showed that LPS‐activated HSCs and downregulated Gpx4 and SLC7A11 in LX‐2 and T6 cells.

### 3.2 Dan attenuated LPS‐induced HSC activation and reversed the downregulation of Gpx4 and SLC7A11 in LX‐2 cells

**FIGURE 2:**
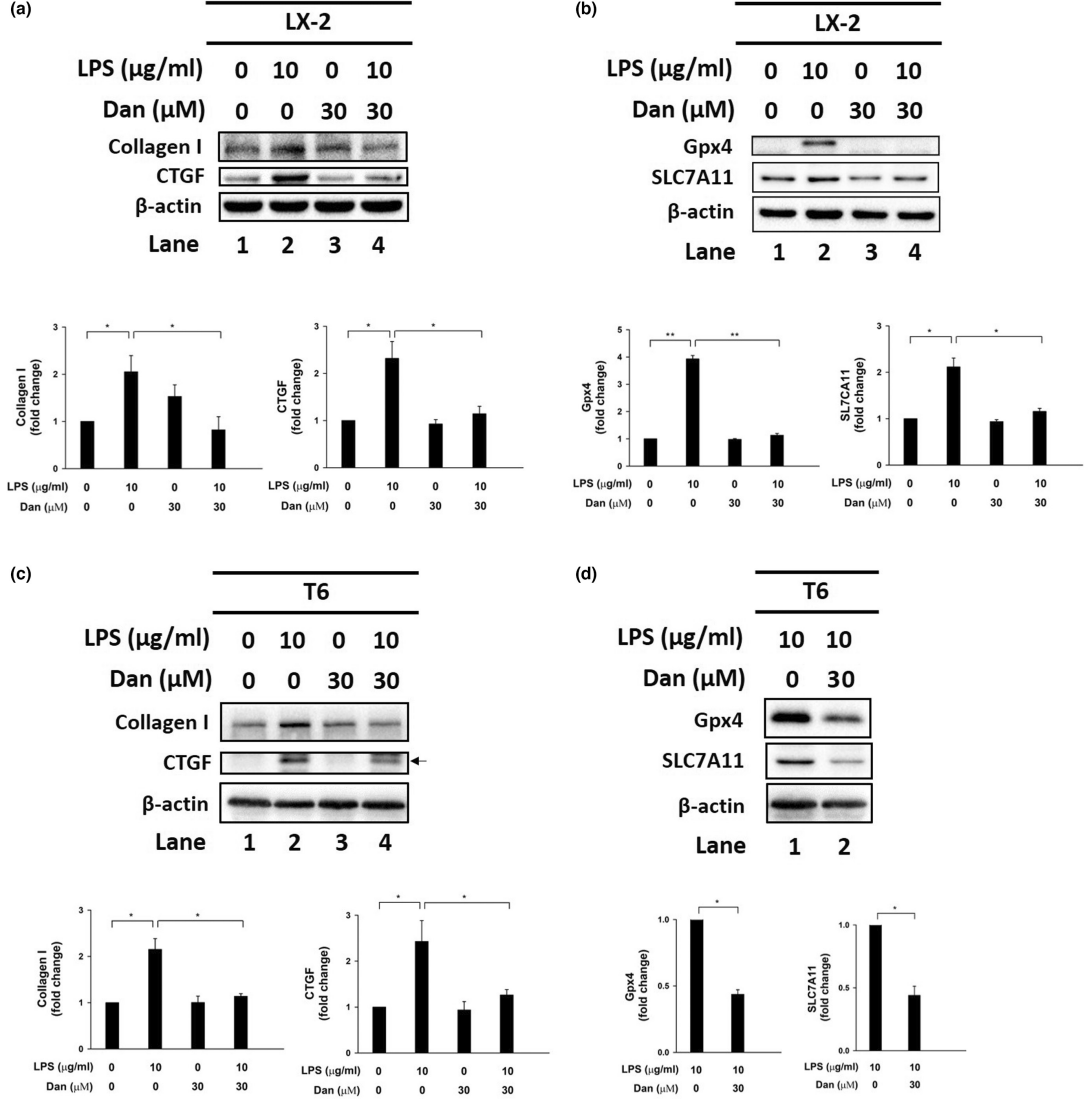
Effect of Danshensu (Dan) on LPS‐induced LX‐2 and T6 cells activation and expression of Gpx4 and SLC7A11. (a) Changes in the protein levels of collagen I and CTGF after LPS treatment or not and in the presence or absence of Dan in LX‐2 cells. (b) Changes in the protein levels of Gpx4 and SLC7A11 after LPS treatment or no treatment, and in the presence or absence of Dan in LX‐2 cells. β‐actin is indicated as an internal control. (c) Changes in the protein levels of collagen I and CTGF after LPS treatment or not and in the presence or absence of Dan in T6 cells. (d) Changes in the protein levels of Gpx4 and SLC7A11 after LPS treatment and in the presence or absence of Dan inT6 cells. β‐actin is indicated as an internal control. Lower panel showed the quantitative analysis of specific proteins using ImageJ. The error bars are represented as standard deviation from three independent replicates. *N* = 3. *p < .05. **p < .01

To determine the role of Dan in the regulation of HSCs, we detected the indicated proteins by western blotting. The results showed that Dan (30 μM) reversed the increased expression of collagen I and CTGF (Figure 2a, lane 2 vs. 4) as well as downregulated Gpx4 and SLC7A11 in LPS‐treated LX‐2 cells (Figure 2b, lane 2 vs. 4). Similar results were shown in LPS‐treated T6 cells (Figure 2c and d). The quantitative results were also shown in the lower panels in each figure (Figure 2, lower panel). Therefore, we suggest that Dan attenuates LPS‐induced HSCs activation through the regulation of Gpx4 and SLC7A11 in LX‐2 and T6 cells.

### 3.3 Dan increased the accumulation of lipid ROS and reversed the upregulation of LCN2 in LPS‐activated LX‐2 cells

**FIGURE 3:**
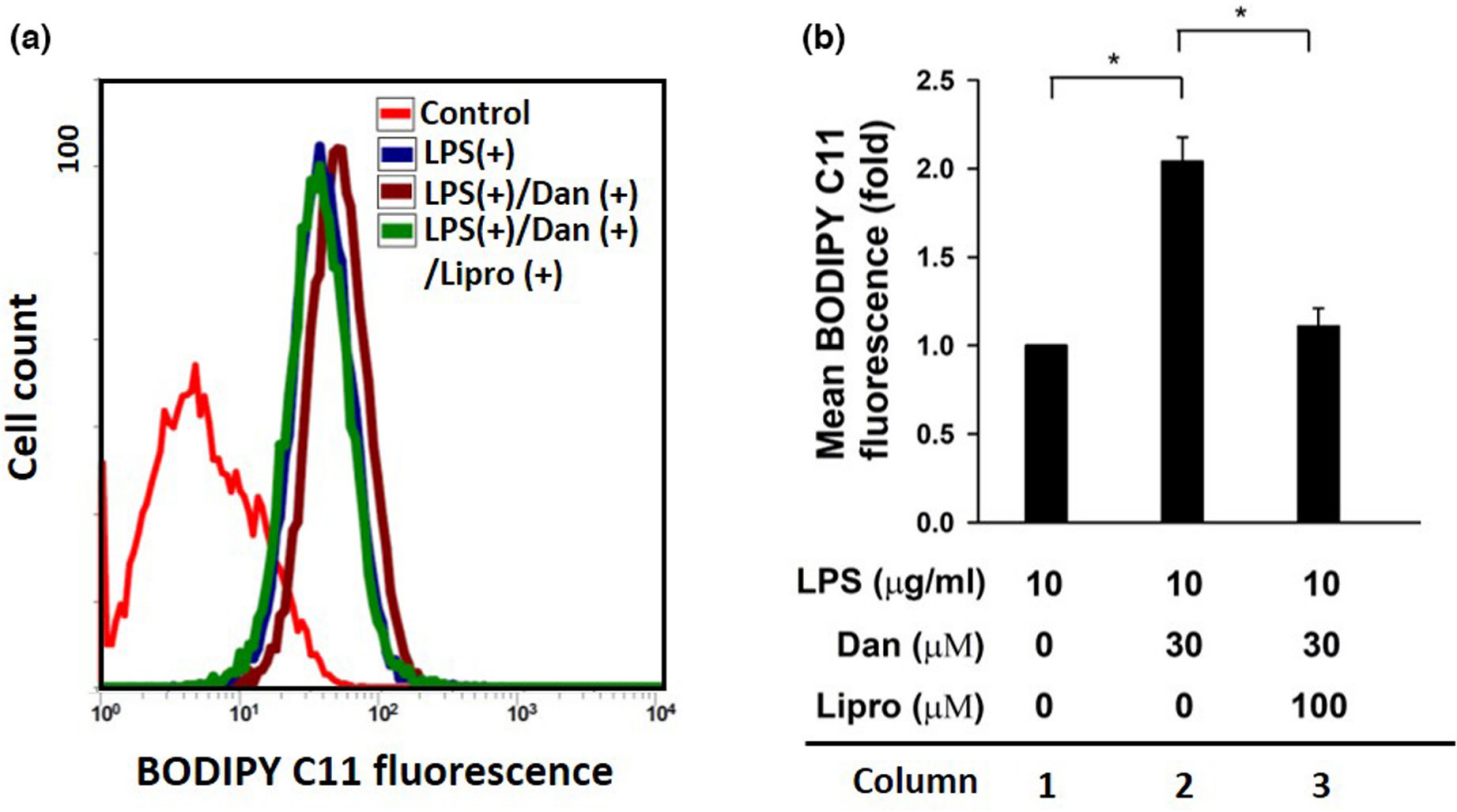
Danshensu (Dan) alleviating LPS‐induced reactive oxygen species (ROS) accumulation in a ferroptosis‐dependent manner. (a) Changes in cellular lipid ROS level of control (without 2 μM C11‐BODIPY staining), treated with 10 μg/ml LPS in the absence of 30 μM Dan (LPS+), in the presence of 30 μM Dan (LPS+/Dan+), or in the presence of 30 μM Dan and 100 μM Lipro (LPS+/Dan+/Lipro+) in LX‐2 cells. (b) The level of lipid ROS is quantified as the mean fluorescence intensity. Error bars represent the standard deviation of three independent replicates. *N* = 3. *p < .05

**FIGURE 4:**
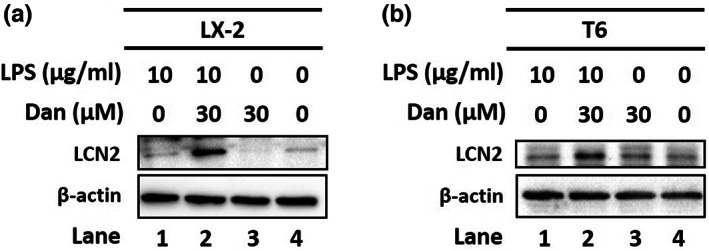
Danshensu (Dan) reversed the upregulation of lipocalin‐2 (LCN2) in LPS‐activated LX‐2 and T6 cells. Changes in the protein levels of LCN2 after LPS treatment or no treatment and the presence or absence of Dan after β‐actin are indicated as an internal control

To further confirm the ferroptotic effects of Dan on LPS‐activated LX‐2 cells, we analyzed lipid ROS levels using BODIPY C11 fluorescence staining. Results showed that Dan significantly induced the production of lipid ROS in LPS‐activated LX‐2 cells (Figure 3a,b, column 1 vs. 2). Furthermore, liproxstatin (Lipro) has been reported as an inhibitor of Gpx4 and prevents iron‐induced oligodendrocyte ferroptosis in oligodendrocytes(Fan et al., 2021). Our data showed that Lipro (100 μM) significantly inhibited the increase in lipid ROS accumulation in Dan and LPS LX‐2 cells (Figure 3a,b, column 2 vs. 3). However, in LX‐2 cells, LPS caused the upregulation of LCN2 (Figure 4, lane 1 vs. 2), which was reversed after treatment with Dan (Figure 4, lane 2 vs. 4). Taken together, our findings suggest that Dan inhibits LPS‐activated HSCs by regulating lipid ROS, ferroptosis, and LCN2.

## 4 DISCUSSION

Inhibition of HSCs activation is effective in preventing and treating liver fibrosis. Evidence indicates that HSCs ferroptosis is induced by berberine induction of ferrous redox in liver fibrosis in LX‐2 cells and thioacetamide‐induced mice models (Yi et al., 2021). Yuan et al. (2022) reported that sorafenib inhibits liver injury and fibrosis through HIF‐1α/SLC7A11 signaling, leading to HSCs ferroptosis. Meanwhile, Kuo et al. (2020) proposed that chrysophanol, isolated from the Chinese herb *Dahuang*, can induce HBx‐induced hepatic fibrosis through ferroptosis and prevent the build‐up of lipid ROS that leads to hepatic fibrosis. Magnesium isoglycyrrhizinate (MgIG)‐induced antifibrotic effects are attributed to ferroptosis in hepatic fibrosis, and the underlying mechanisms of ferroptosis and MgIG‐induced antifibrosis depend on heme oxygenase‐1‐mediated ferroptosis (Sui et al., 2018). The process of ferroptosis in liver fibrosis remains unclear, despite contradictory findings (Yu et al., 2020; Zhang et al., 2020). It is unclear what role ferroptosis plays in reducing liver fibrosis via Dan‐mediated mechanisms.

In this study, we suggested that Dan attenuates LPS‐induced HSCs activation by regulating ferroptosis in LX2 and T6 cells. Ferroptosis is triggered by tripartite motif‐containing protein 26, which inhibits liver fibrosis and hepatic steatosis by ubiquitinating SLC7A11 (Zhu et al., 2021). However, a CCl4‐induced liver fibrosis animal model inhibiting xCT/SLC7A11 exacerbates chronic liver damage while killing myofibroblastic HSCs in the liver (Du et al., 2021). In our study, Dan reversed LPS‐induced SLC7A11 upregulation in LX‐2 cells. Therefore, further evidence is required to confirm the role of Dan in ferroptosis in a SLC7A11‐dependent manner in vivo and in vitro.

Lipocalin‐2 undergoes transactivation to induce ferroptosis resistance by transactivating nuclear protein 1, a stress‐inducible transcriptional regulator, which may be the driving force behind ferroptosis resistance (Liu et al., 2021). In xenograft tumors derived from hepatocellular carcinoma patients with low LIF receptor subunit alpha expression and high expression of LCN, an LCN2‐neutralizing antibody enhances the ferroptosis‐inducing and anticancer effects (Yao et al., 2021). Ethanol‐fed SIRT1 KO mice have also been reported to produce less LCN2, reflecting factors such as iron deprivation, attenuated neutrophilia, and diminished ferroptotic damage to the liver (Zhou et al., 2020). In this study, the results indicated that Dan reversed LPS‐upregulated LCN2 expression in LX‐2 and T6 cells. Therefore, targeting LCN2 might improve liver fibrosis by regulating ferroptosis.

## 5 CONCLUSIONS

**FIGURE 5:**
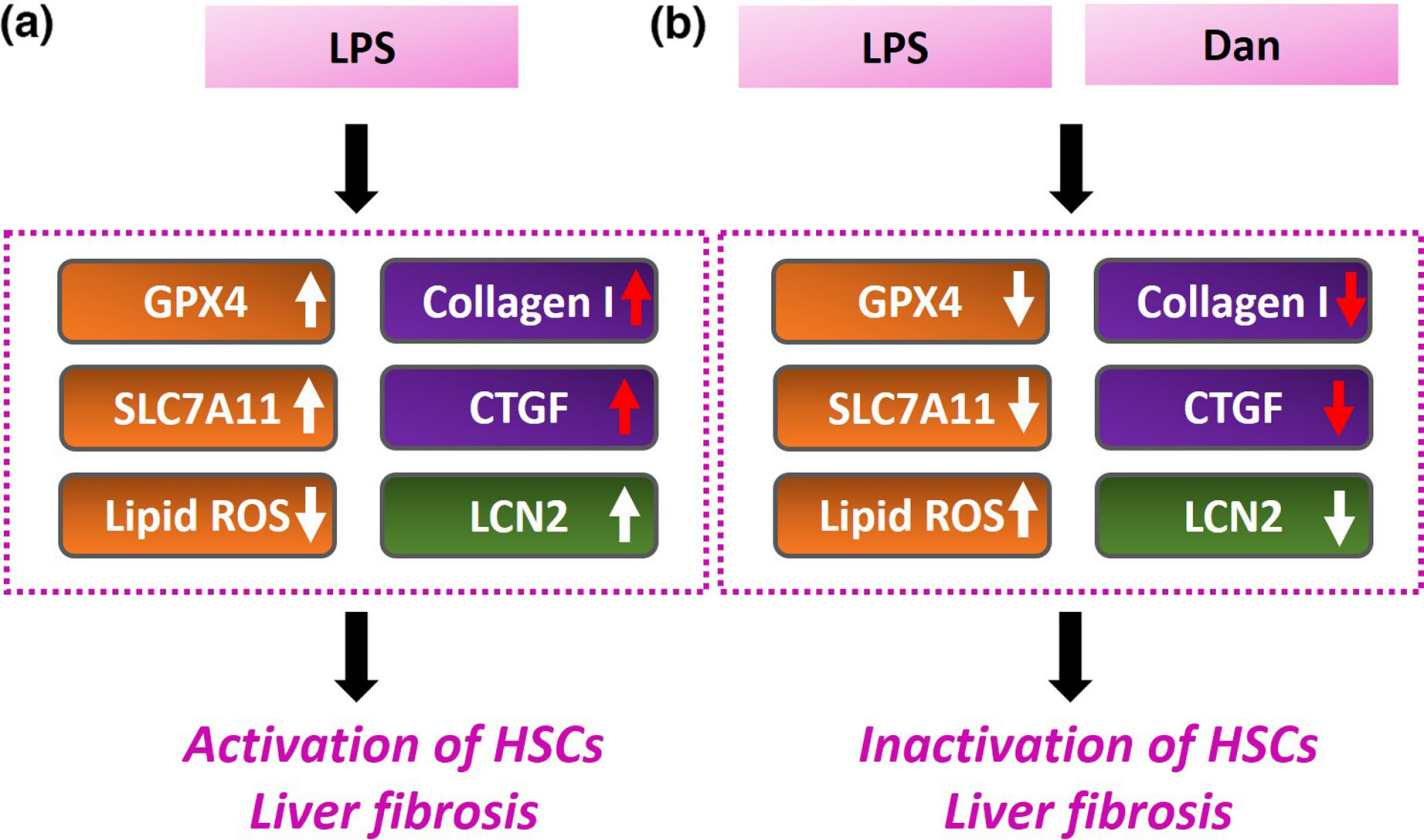
This schematic illustrates the functional mechanisms by which Danshensu (Dan) influences HSCs activated by LPS. (a) LPS triggered the HSCs activation via upregulation of GPX4, SLC7A11, collagen I, CTGF, and LCN2 as well as decrease in ROS accumulation. (b) Dan reversed LPS‐induced HSCs activation via downregulation of GPX4, SLC7A11, collagen I, CTGF, and LCN2 as well as increase in ROS accumulation

Our results showed that Dan attenuated the activation of LPS‐induced HSCs by decreasing the expression of collagen І, CTGF, Gpx4, SLC7A11, and LCN2 as well as increasing the accumulation of lipid ROS (Figure 5).

## FUNDING INFORMATION

Academic funding from the Second Affiliated Hospital of Fujian Medical University (Serial No. BS201902).

## CONFLICT OF INTEREST

The authors declare that they have no conflicts of interest with respect to the publication of this article.

## Data Availability

Data used to support the findings of this study have been included in this article.
